# Chemical Shift-Encoded MRI of Bone Metabolic Markers in Ankylosing Spondylitis

**DOI:** 10.1155/2022/1846667

**Published:** 2022-10-13

**Authors:** Li-ping Ma, Cui-yun Sheng, Long Qian, Zhi-bin Zeng, Gen Li

**Affiliations:** ^1^Department of Radiology, Huazhong University of Science and Technology Union Shenzhen Hospital, Shenzhen, Guangdong, China; ^2^MR Research, GE Healthcare, Beijing, China; ^3^Department of Emergency, Huazhong University of Science and Technology Union Shenzhen Hospital, Shenzhen, Guangdong, China

## Abstract

**Objective:**

To investigate the feasibility and correlation of sacroiliac joint (SIJ) fat fraction (FF) and R2^∗^ as markers of bone metabolism in patients with ankylosing spondylitis (AS).

**Methods:**

75 AS patients were classified into an early active group (EA), late active group (LA), and inactive group (IA). Additionally, 54 matched healthy individuals were selected to be part of the normal control group (NC). All participants underwent chemical shift encoded based MRI (IDEAL-IQ) and routine clinical SIJ MRI at 3.0 T. FF and R2^∗^ were measured in subchondral bone, bone marrow edema (BME), and fat metaplasia (FM). Out of the participants, 39 with BME lesions (15 from EA, 16 from LA, 8 from IA) and 39 with FM lesions (9 from EA, 17 from LA, 13 from IA) were included. Differences in FF, R2^∗^ value for subchondral bone of all participants and for BME, FM lesions were evaluated. Subsequently, different stages of BME and FM in patient groups were compared, and the relationship between FF and R2^∗^ was analyzed.

**Results:**

A significant difference in FF was demonstrated among the BME, FM and the normal bone marrow (*p* < 0.001), meanwhile, the difference of R2^∗^ value in FM was significantly lower (p = 0.034, 0.012) than that of BME and that of normal bone marrow. At lever of different lesions, only the FF for BME was significantly different among 3 patient groups (*p* = 0.001), while there was no significantly different FF for FM among 3 patient groups. Unlike in BME lesions, the FF in FM lesions had a negative correlation with R2^∗^ (*p* < 0.001, *r* = −0.488).

**Conclusion:**

FF and R2^∗^ measurements help to quantitatively analyze the bone marrow fat composition and bony trabecular microstructure changes in AS, providing a noninvasive and accurate assessment basis for AS bone metabolism abnormalities.

## 1. Introduction

Ankylosing spondylitis (AS) is a chronic spondyloarthropathy that occurs frequently in adolescents, with insidious onset and relapsing nature, characterizing irreversible pathological progressive disease from inflammation, bone destruction to the formation of new bone [1, 2]. Bone formation may occur in parallel with bone mineral content loss in AS [3, 4]. Syndesmophyte formation and progressive ankylosis as well as fracture caused by bone loss are main causes of disability. Ignoring bone metabolism abnormalities in the management of AS exposes the patient to impaired quality of life, limited functioning, even severe emotional and mental conditions, particularly for young patients deprived of workability. Bone marrow adipose tissue, as an important immune tissue, is thought to be involved in the regulation of bone metabolism [5]. Early detection and quantitative monitoring of bone metabolic abnormalities may help to avoid advanced joint fusion and improve the prognosis. However, the relationship between inflammation, fat, and bone metabolism abnormalities in the pathogenesis of AS is elusive.

Despite the fact that Dual-energy X-ray absorptiometry (DXA) has been the most widely available bone densitometry method, DXA is not a true volumetric measure of bone marrow density, but rather a projection “areal” (g/cm 2) measurement of whole bone, so it is influenced by the presence of degenerative changes in the spine, scoliosis, arteriosclerosis in the abdominal aorta, other abdominal calcifications, and fat [6]. Therefore, it may be beneficial to investigate an alternative quantitative measurement with good accuracy and ability to detect small changes, especially for those who are not exposed to ionizing radiation.

The chemical-shift-encoded (CSE) imaging sequence is an exceedingly available and considering quantitative assessment of changes in bone density by bone marrow fat content [7], with clear advantages of higher spatial resolution and no-ionizing radiation over traditional bone densitometry method DXA, which is of utmost importance in the typically young patient population. The quality of the CSE-MRI sequence has substantially improved since 1984. Recently, IDEAL-IQ sequence, as an advanced multi-echo CSE-MRI method, use multiecho techniques to correct for T2^∗^ decay with a multipeak fat model to accurately quantify the FF [8]. Simultaneously, T2^∗^ measurement is sensitive to inhomogeneities caused by susceptibility differences at the interface between bone marrow and trabecular bone, allowing us to estimate bone status from the same CSE-MRI data. The use of MR techniques to obtain information on the properties of bone provides an important tool for identifying the microarchitectural characteristics of bone in researches of osteoporosis [6, 9]. Patients with osteoporosis have prolonged T2^∗^ decay in the bone marrow (i.e., reduced decay rate *R*2^∗^ = 1/*T*2^∗^), likely due to decreased microscopic susceptibility from trabecular bone [6]. Therefore, R2^∗^ may provide a measure of new bone formation and bone destruction, both of which are key processes in AS [10] . Thus, this study was to assess the feasibility and correlation between sacroiliac joint (SIJ), fat fraction (FF), and R2^∗^ as markers of bone metabolism in patients with AS.

## 2. Materials and Methods

### 2.1. Subjects

This was a cross-sectional analysis approved by the clinic's ethics board of Huazhong University of Science and Technology Union Shenzhen Hospital with written informed consent was obtained from all participants. All patients ≤50 years of age with fulfilled AS Assessment of the modified New York Criteria (*n* = 75; 43 male; mean age 33.83 ± 7.20 years; range 15 to 50 years) were consecutively enrolled from the Rheumatology Department from October 2020 to November 2021. Moreover, 54 healthy people (27 male; mean age 34.05 ± 7.51 years; rang 20 to 48 years) were recruited to form a normal control group (NC). Participants with tumors, infectious lesions, fractures, or surgical history as well as those with metabolic diseases, and those using medications, such as long-term oral glucocorticoids, long-term oral heparin, antiepileptic drugs, methotrexate, cyclosporine, and affect bone metabolism were excluded from this study.

All patients were categorized into three groups: 49 active patients with a Bath Ankylosing Spondylitis Disease Activity Index (BASDAI) score of >6 (on a 0–10 scale) or BASDAI score of 4 to 6 and erythrocyte sedimentation rate (ESR) ≥20 mm/h (Westergren) or C-reactive protein (CRP) ≥6.0 mg/L, others (26) were classified into the inactive group (IA). Then, 24 of 49 active patients without treatment with drug therapy and no radiologic sacroiliitis (Sacroiliitis not seen on radiographs) were subdivided into an early active group (EA), while the remaining 25 active patients were then divided into a later active group (LA). Clinical indicators, such as age, gender, disease duration, BASDAI scores, and laboratory indexes for active inflammation (ESR and CRP) were collected. Participants' demographic and clinical characteristics are shown in Table 1.

### 2.2. MRI Acquisition and Postprocessing

MR images were performed using a 3.0 T MRI system (SIGNA Pioneer, GE Healthcare, Milwaukee, WI) with a 16-channel phased array body coil. The MRI protocol included the routine oblique coronal T1WI, T2WI, T2-weighted, and fat-saturated (T2WI-FS) as well as oblique axial IDEAL-IQ sequence performed in an oblique axial plane along the axis of SIJ to obtain details of lesions with TR: 10.7 ms; TE: 1 ms; FOV: 300 × 300 mm; matrix: 152 × 152; flip angle: 4 degrees; slice thickness: 3 mm. A total 34 slices were scanned within 128 seconds. (Table 2).

**Figure 1:**
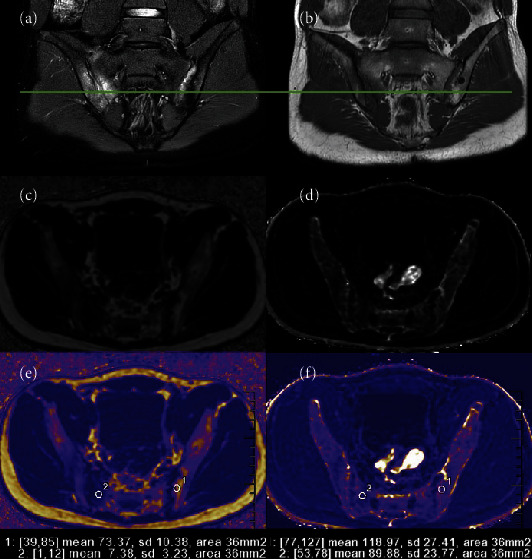
A 17-year-old male AS patient from late active group (LA). T2WI-FS and T1WI (a and b) Focal irregularity FM along the entire left iliac, and extensive BME was presented on right sacrum. FF maps (c and e) and R2^∗^ maps (d and f) demonstrated and measure ROI-1 of FM and ROI-2 of BME. AS: ankylosing spondylitis; BME: bone marrow edema; FM: fat metaplasia; FF: fat fraction; ROI: region of interest.

For IDEAL parameters analysis, all data were transferred to workstation (ADW4.7, GE Healthcare) automatically creating a water phase, fat phase, in-phase, opposed-phase, R2^∗^mapping, and FF mapping. Definitions of lesions of the SIJ on MRI for BME and FM, which were adopted according to the Assessment of SpondyloArthritis international Society (ASAS). BME in the SIJ was evaluated on T2WI-FS sequence and must be clearly presented proximity to the subchondral bone on at least 2 consecutive slices. The appearance of postinflammatory FM has characteristic features defined by a distinct border, homogeneous increase in T1W signal and extended more than 1 cm away from the SIJ surface. The FF and R2^∗^ data were independently evaluated by two observers (L.P.M., Reader A, and L.Y.S., Reader B with 4 and 7 years of working experience with musculoskeletal MR imaging, respectively) blinded to patient demographics, in order to eliminate the influence of subjective bias. Decision was made in consultation with an adjudicator (Z.B.Z. with 27 years diagnostic experience in musculoskeletal MR imaging). An elliptic region of interest (ROI) of approximately 20-50 mm2 was placed in each BME lesion at its maximum transverse level, on the FF mapping and the R2^∗^ mapping, avoiding the bone cortex, and articular cartilage (Figure 1). ROI values of BME lesions were measured thrice and averaged. We then adopted this same approach to measure the FF and R2^∗^ in FM lesions. For all participants including the control group, ROIs were also drawn on each side of the juxta-articular bone marrow to provide internal standardization, and we defined the mean value of SIJ's subchondral bone in the control group as normal bone marrow.

### 2.3. Statistics Analysis

All variables of clinical data and MRI parameters were analyzed using the Statistical Product and Service Solutions (SPSS) software (version 22.0; IBM, Armonk, NY) and described as mean ± standard deviation or as a percentage (%). It was assumed that distributions of FF (the unit is a percentage) were not normal; the Kolmogorov-Smirnov and Levene's test were used to test for normality and homoscedasticity for other variables. R2^∗^ value of subchondral bone and lesions follow normal distribution. For normally distributed data, one-way ANOVA was used to compare differences among groups. For nonnormally distributed data, the Kruskal-Wallis test was used. Then, post hoc analysis was used to evaluate differences between each two groups. The degree of association among FF, R2^∗^, and CRP was calculated using the Spearman correlation coefficient (*r*). The level of significance was set at *p* < 0.05.

## 3. Results

The clinical characteristics of the patients and the NC included in the study are summarized in Table 1. The Symptom duration and CRP were significant difference among 3 patient groups (*p* < .001, *<*0.001, respectively). However, neither the FF nor R2^∗^ value for subchondral bone of the SIJ delineated any difference across the 4 groups.

### 3.1. Differentiation of FF and R2^∗^ Value Based on the Affected BME, FM, and Normal SIJ's Subchondral Bone

A significant difference in FF was demonstrated among the BME, FM, and the normal bone marrow (*p* < 0.001); the average FF were 30.62% ± 18.48%, 84.00% ± 8.66%, 59.32% ± 9.27%, respectively. Meanwhile, there was a significant difference in R2^∗^ value among the BME, FM, and the normal bone marrow (*p* = 0.016); the difference of the mean R2^∗^ value in FM lesions was significantly lower (*p* = 0.034, 0.012) than that of BME lesions and that of normal bone marrow, but no significant difference in R2^∗^ value between BME and the normal bone marrow (*p* = 0.14) (Table 3).

### 3.2. Differentiation of FF and R2^∗^ Value on the Different Stages

The frequency of BME lesions in IA was significantly different from EA and IA (*p* = 0.031). The proportion of fat deposition in the BME lesions was the highest in IA (*p* = 0.001), followed by LA, among the 3 patient groups, but the difference in the FF of BME lesions between the two active groups was no statistical difference (*p* = 0.491). Whereas, there was no difference in the R2^∗^ value of BME lesions among the 3 groups (*p* = 0.146).

In FM lesions, there was no significant difference among 3 groups in the frequency of FM lesions (*p* = 0.112). No difference was observed in neither the FF nor R2^∗^ value in FM lesions among 3 patient groups.

### 3.3. Correlations between FF, R2^∗^, and CRP in Lesions

**Figure 2:**
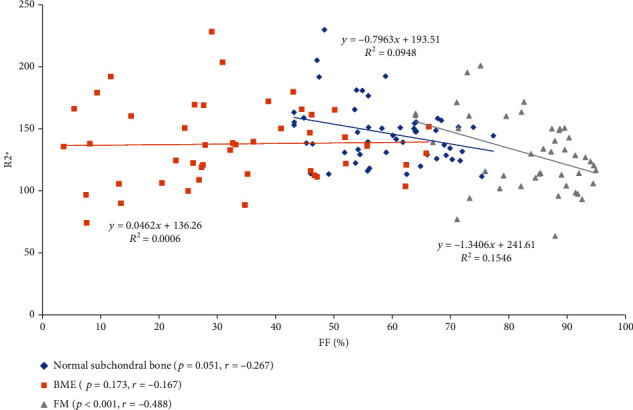
Correlation plots between fat fraction (FF) and R2^∗^ value in the bone marrow edema (BME), fat metaplasia (FM) lesions and normal juxta-articular bone marrow.

**Figure 3:**
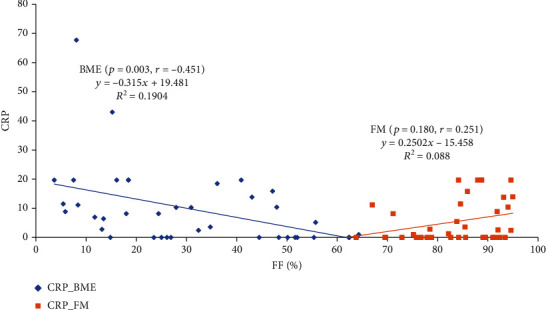
Correlation plots between fat fraction (FF) and C-reactive protein (CRP) in the bone marrow edema (BME) and fat metaplasia (FM) lesions in the patients with ankylosing spondylitis (AS).

The Spearman correlation analysis revealed that FF correlated inversely with R2^∗^ for FM lesions (*p* = 0.000, *r* = −0.488). There was no significant correlation between FF and R2^∗^ for BME lesions (*p* = 0.173, *r* = −0.167), while an increase in the CRP value for BME lesions displayed a negative correlation with a decrease in FF for BME lesions (*p* = 0.003, *r* = −0.451). There was no significant correlation between a change in FF for FM lesions and CRP (*p* = 0.180, *r* = 0.251) (Figures 2 and 3).

## 4. Discussion

Here, we present a noninvasive quantitative approach to the assessment of bone marrow component and microstructure changes in AS using CSE-MRI. We found that FF could accurately reflect the changes of bone marrow components in BME and FM regions, and further observe the increased of FF in EA group after the resolution of inflammation in the BME. Additionally, we show that R2^∗^ are reduced in areas of FM and a negative correlation between FF and R2^∗^, which may be due to changes in bone trabecular structure. CSE-MRI measurements, by combining bone fat component with R2^∗^ to predict bone microstructural characteristics, holds promise as imaging markers for evaluation of bone metabolic abnormities over time.

The presence of periarticular and subchondral BME is a strong criterion for active inflammation, and change of BME is assessed when scoring activity [11]. However, in this study, the resolving inflammatory process observed in LA as a slight hyperintensity on T2WI-FS, may be mainly attributed to the classification criteria set without MRI imaging modalities relying on clinical and laboratory findings alone. Moreover, the FF and R2^∗^ data were evaluated by radiologist blinding in clinical trials. There was a significant difference among patient groups in FF for BME lesions, with mean FF values of 17.05% ± 17.52%, 27.93% ± 19.41%, and 52.05% ± 15.23%, respectively. Moreover, the proportion of fat deposition in FM lesions distributed between 60% and 95% was higher than those of normal bone marrow of the SIJ from the NC group ranging from 40% to 70%. While, the lowest FF in BME lesions was at a range of approximately 0% to 65%. As discussed by Koo et al. [12], suggested FF increase indicates the chronicity of SpondyloArthritis. Previous study [13] also found the FF changes in BME. In this study, an increasing FF in the BME lesions was related to the healing process of osteitis. Furthermore, an elevated level of FF in BME lesions delineated a negative correlation with a decrease in CRP. This supports an earlier hypothesis [1] that bone marrow adipocyte suggesting a healing process is taking place in the affected inflamed periarticular areas.

FM lesions were observed in 9 of the 39 patients in EA (23.08%), suggesting that FM is present in the early course of the disease. There was no significant difference in FF for FM lesions among 3 patient groups. However, 17 of the 39 patients in the LA group (43.59%) and 13 of the 39 in IA group (33.33%) had more profuse/confluent FM lesions, and this could be explained by the LA cohort characterized by long disease duration and high disease activity. Previously, Guo et al. [14] quantitatively estimated the fat portion in the SIJ of AS patients using IDEAL-IQ and observed a decrease in fat/water signal ratios of fat infiltration regions and normal-appearing regions after 6 months of treatment. However, the patients in the IA group had a relatively short course duration, most of them being follow-up patients after drug therapy. The patients had a stable condition, and whether the reduction of FM lesions is related to drug treatment requires further longitudinal observation and research to confirm.

For R2^∗^ measurements, there were no significant differences among groups for neither BME nor FM lesions. However, the R2^∗^ observed in FM lesions was significantly lower than that in BME and normal SIJ bone morrow. Furthermore, there was a negative correlation between the FF and R2^∗^ value in FM lesions, suggesting that changes in the R2^∗^ value occurred in the area of FM lesions, and FF was inversely correlated with bone mass. Previous osteoporosis studies [6, 15, 16] have found that vertebral R2^∗^ in osteoporosis patients is inversely correlated with FF, which is consistent with our findings. There is an inverse relationship between bone marrow adipose tissue and whole-body bone mineral density [17]. The differentiation of adipogenic and osteogenic cell lines are conversely derived from the mesenchymal stem cells [6]. This may be an explanation for the decreasing R2^∗^ values in FM lesions. However, other in vitro studies [15, 18] found that R2^∗^ value might capture the microarchitecture of the bone but may be affected by other marrow component.

There is no significant difference in R2^∗^ changes within the BME lesions, implying that there may be no bone structure change in BME sites of the SIJ. Recently, the relationship between inflammation and bone metabolism generated a considerable debate. Considering the irreversible nature of the inflammatory-sacroiliitis, early adequate management was recommended by the ASAS. Nonetheless, longitudinal studies with drug therapy found that anti-inflammatory therapy in patients with AS apparently fails to impede the progression of AS [19, 20]. On the contrary, some studies [21, 22] detected that either the subchondral infiltrative process in the SIJ or inflammation on spinal MRI is negatively correlated with vertebral bone mineral density in patients with AS. Fat metaplasia is regarded as a critical pathway from inflammation to new bone formation [5], immunology research [23] involving a biopsy of the fat signal detected by MRI in the spine of patients with AS also discovered that MRI fat lesions had more osteoblastic than osteoclastic activity (6.9 vs 0.17 cells/HPF). In a longitudinal study of AS, Kang et al. [24] found that vertebrae with both FM on MRI and low bone density at baseline tended to have more new bone formation 2 years later, while 32% of new bone formation occurred at vertebrae with no FM at baseline, and indicated that inflammation-driven changes in the microarchitecture affected the mechanical properties of the spine and could trigger bone repair at anatomically distinct sites to maintain biomechanical stability. In our study, there was no change in the microstructure of SIJ BME lesions. This discrepancy may be attributed to both differences in the skeletal properties of the measurement sites and biomechanical factors. Most previous measurements were generally applied to the lumbar spine for clinically evaluating bone loss and semiquantitative assessment of modified Stoke Ankylosing Spondylitis Spinal Score for new bone formation. As far as we know, few previous research has evaluated structural changes of the SIJ. Since the SIJ is one of the largest axial joints with lots of strong ligaments to maintain its position, weight-bearing and pressure distribution, the SIJ has a more complex trabecular structure in terms of biomechanics.

This study has several limitations. First, this study was limited to the subchondral bone, BME, and FM lesions of the sacroiliac joint and did not study the changes in articular cartilage and spinal vertebral body. Endochondral ossification and ectopic cartilage ossification of ligaments lead to joint fusion and syndesmophytes formation. Therefore, future research should comprehensively evaluate whole-body bone changes, especially the vertebrae as well as changes in articular cartilage ossification. Second, progressive partial ankylosis, as structural lesions, is detected best in follow-up studies to understand their origin and association with active lesions. Third, we did not analyze the effects of drug therapy on bone structure changes. Fourth, the IDEAL was not performed in some orientation/slice thickness with T1WI and T2WI-FS. Last, further studies are needed to compare the diagnostic accuracy with other quantitative bone measurements, such as quantitative computed tomography as well as biopsy pathology studies for confirmation.

## 5. Conclusions

In summary, the FF within the BME lesions of the SIJ demonstrated that the resolution of inflammation was accompanied by an increase in FF, and the FF and R2^∗^ values in the FM lesions were inversely correlated. Meanwhile, the R2^∗^ value for FM lesions was the lowest, compared with BME lesions and normal SIJ bone marrow. The IDEAL-IQ technique allows to simultaneously estimate the FF and R2^∗^ in the SIJ, which can accurately measure the fat content and reflect the microstructural changes in the bone marrow. As a systemic metabolic bone disease, there is a need to perform a more comprehensive assessment of the changes in bone metabolism throughout the body in further studies on AS to provide MRI-based evidence for recommendations regarding the monitoring of inflammation and structural damage and in the identification of the pathogenesis of ankylosis and osteoporosis.

## Figures and Tables

**Table 1:** Comparation of demographic characteristics, clinical and imaging variables in the AS study population ($\bar{x\;}$±s).

Parameters	All patient	Early active group (EA)	Late active group (LA)	Inactive group (IA)	Normal control group (NC)	*P*
	(*N* = 75)	(*N* = 24)	(*N* = 25)	(*N* = 26)	(*N* = 54)	
Ag e(y)	31.16 ± 7.88	30.96 ± 8.11	29.88 ± 7.42	32.58 ± 8.17	34.05 ± 7.51	>0.050
Symptom duration (y)	4.21 ± 4.70	1.66 ± 1.13	7.05 ± 5.93	3.94 ± 4.14		<0.001
CRP (mg/L)	8.30 ± 10.57	16.29 ± 11.97	7.74 ± 4.45	1.69 ± 8.27		<0.001
Subchondral bone FF (%)	57.74 ± 16.25	51.12 ± 20.41	64.40 ± 15.57	57.71 ± 9.35	59.32 ± 9.27	>0.050
Subchondral bone R2^∗^	149.90 ± 29.09	148.77 ± 22.85	148.09 ± 25.51	152.40 ± 36.53	146.27 ± 23.96	>0.050
BME lesions	39	15 (38.46%)	16 (41.03%)	8 (20.61%)		0.031
BME FF (%)	30.62 ± 18.48	17.05 ± 17.52	27.93 ± 19.41	52.05 ± 15.23		0.001
BME R2^∗^	138.57 ± 33.18	145.23 ± 33.25	136.06 ± 37.12	122.25 ± 12.93		0.146
FM lesions	39	9 (23.08%)	17 (43.59%)	13 (33.33%)		0.112
FM FF (%)	84.00 ± 8.66	87.95 ± 9.01	85.44 ± 8.29	84.19 ± 9.25		0.852
FM R2^∗^	123.05 ± 29.40	123.68 ± 33.99	127.01 ± 30.94	103.17 ± 22.31		0.248

AS: ankylosing spondylitis; BME: bone marrow edema; CRP: C-reactive protein; FF: fat fraction; FM: fat metaplasia.

**Table 2:** Parameters of routine SIJ MRI sequences and IDEAL-IQ in the AS study.

Parameter	T1WI	T2WI	T2WI-FS	IDEAL-IQ
Sequence	Fast spin echo	Fast spin echo	Fast spin echo	Gradient recalled echo
Imaging plane	Oblique coronal	Oblique coronal	Oblique coronal, oblique axial	Oblique axial
Repetition time (msec)	514	3213	2544, 2500	10.7
Inversion time (msec)	Minimum	112	68, 65	1
Field of view (mm)	260 × 260	260 × 260	260 × 260, 300 × 300	300 × 300
Matrix(frequency × phase)	352 × 256	352 × 265	352 × 256, 320 × 256	152 × 152
Flip angle (degrees)	111	111	142, 142	5
Slice thickness/gap(mm)	3.5/0.5	3.5/0.5	3.5/0.5, 3.5/1.0	3.0/0
Number of excitons	1	1.5	1.25	2
Echo train length	4	24	14,14	3
Bandwidth (kHz)	62.5	35.71	31.25, 41.67	142.86
Total slices	20	20	20, 20	34
Acquisition time(s)	89	110	153, 150	128

SIJ: sacroiliac joint; AS: ankylosing spondylitis.

**Table 3:** Comparation of fat fraction (FF), R2^∗^ value in different areas of sacroiliac joint in patients with AS ($\bar{x\;}$±s).

Parameters	BME	FM	Normal subchondral bone	*P*
	(*N* = 39)	(*N* = 39)	(*N* = 54)	
FF (%)	30.62 ± 18.48	84.00 ± 8.66	59.32 ± 9.27	<0.001
R2^∗^	138.57 ± 33.18	123.05 ± 29.40	146.27 ± 23.96	<0.016

AS: ankylosing spondylitis; BME: bone marrow edema; FF: fat fraction; FM: fat metaplasia.

## Data Availability

The datasets used and/or analyzed during the current study are available from the first author Li-ping Ma (lpma1106@163.com) on reasonable request.

## Supplementary Materials

Supplementary Materials Table S1: overview of FF, R2^∗^ values and clinical data of 4 groups. Table S2–S5: FF and R2^∗^ value of each ROI in different 4 groups.
