# Supplementary material for: Chemical Shift-Encoded MRI of Bone Metabolic Markers in Ankylosing Spondylitis
Source: Dis Markers. 2022 Oct 13;2022:1846667. doi: 10.1155/2022/1846667 (PMC9584712; doi:10.1155/2022/1846667)
Supplement: Supplementary Materials — Table S1: overview of FF, R2∗ values and clinical data of 4 groups. Table S2–S5: FF and R2∗ value of each ROI in different 4 groups. [file 1846667.f1.zip › Table S1.pdf]

Data for bone metabolic study in the patients with AS

Name	Age	Sex	Group	SCB_FF	SCB_R2*	BME_FF	BME_R2*	FM_FF	FM_R2*	CRP	HLA-B27	ESW	Date
宁盼盼	30	F	EA	56.05	173.71	25.86	122.44	88.76	150.05	16.7	630		2021/4/29 14:17
廖秋妹	34	F	EA	53.54	163.05	0		82.17	163.52	1.28	672	11	2021/6/3 16:24
夏水兰	44	F	EA	17.55	98.74	11.73	192.09	0		6.97	712	12	2021/6/2 17:38
杨小莉	30	F	EA	63.32	121.21			84.16	97.15	20	630		2021/6/1 16:26
陈韵怡	30	F	EA	70.84	168.93	44.48	165.75	88.64	148.95	13.4	62		2021/6/1 10:19
吴旭基	27	M	EA	58.29	148.23	46.69	112.57	88.5	95.87	4.66	630	8	2021/7/17 9:04
陈庆鸿	50	M	EA	84.76	151.05	50.15	165.32	94.02	123.68	10.4	919	69	2021/7/16 7:27
刘新火	22	M	EA	54.01	185.56	9.41	179.15	91.45	98.55	67.7	716	107	2021/7/13 9:33
宋艺敏	25	F	EA	48.42	137.85	27.31	119.11	90.35	142.83	20.3	630		2021/7/10 10:01
钟杰	28	M	EA							13.1	630		2021/7/7 9:42
彭欲娴	33	F	EA	53.13	163.96	3.67	135.64			18.1	630		2021/8/12 0:00
谭珊珊	33	F	EA	58.26	115.95	45.89	146.89			12.7	58		2021/8/11 0:00
王慧敏	29	F	EA	57.12	190.08	24.43	150.67			21.6	48.3	2	2021/9/3
祖靖	44	M	EA	61.11	139.02					11.7	151	2	2021/9/1
卢健	31	F	EA							13	50.7		2021/10/2 8:11
宋梓闻	11	M	EA	44.37	179.05	38.76	172.14			18.5	689	63	
沈睦	41	M	EA	70.62	132.77	46.19	161.39	87.95	63.57	20.7	663		2021/7/13 9:03
谢远仙	26	F	EA	72.76	130.58	0		0		12.6	630		
阮美君	24	F	EA	48.98	146.32	7.5	96.8	71.08	171.37	19.7	630		2021/10/26 9:10
黄雨湘	32	M	EA	53.62	151.94	20.5	106.36	71.14	77	8.16	685	32	2021/1/27 14:59
王佳	31	M	EA	56.98	153.14	27.67	169			16.7	47.5	13	2021/2/1 9:29
冯桂萌	26	F	EA	61.54	138.07	22.91	124.47			26.2	43.6		2021/3/3 10:12
郭冬琪	26	F	EA	42.98	133.81	13.48	89.95			6.43	978	16	2021/3/26 11:05
刘维平	36	F	EA	38.72	149.99	8.15	137.98			7.19	81.3		2021/3/26 14:52
吴国龙	28	M	LA	58.44	161.06	13.15	105.65			2.79	negative	10	2021/2/1 11:05
申三玉	28	M	LA	54.17	159.37	35.19	113.53	94.6	120.48	2.43	803	24	2021/4/25 14:53
程思	21	M	LA	82.88	151.77	43.03	179.84	93.14	127.01	13.9	566	68	2021/4/6 13:00
蔡钦童	32	M	LA	61.46	191.79	5.45	166.17	84.57	110.47	11.5	679	59	2021/5/26 15:37
罗可美	27	F	LA	53.21	176.16			92.03	114.22	2.58	112	40	2021/5/14 15:48
洪美凤	31	F	LA	44.99	164.96	26.1	169.45			0		18	2021/5/10 14:12
肖亮	30	M	LA	77.04	138.13			78.45	101.82	2.84		67	2021/6/25 17:54
叶和兴	32	M	LA	77.86	115.29	27.54	120.86	92.58	93.15	6.2	587	54	2021/6/21 14:33
何联	38	M	LA	65.26	155.43	47.12	111.32	85.94	133.89	15.9	709	57	2021/6/5 12:24

凌春强	27	M	LA	69.24	165.02					10.3	41.8		2021/7/30 8:41
余琼花	52	F	LA	68.98	136.03	55.7	136.06	79.56	160.24	5.16	816	59	2021/7/28 14:39
曾雪涓	31	F	LA	55.85	157.99	27.93	136.89			10.3			2021-08-31
罗惠	21	F	LA	75.39	122.36	46.02	116.04	91.84	97.5	8.86		47	2021/8/19 0:00
林鹏彬	37	M	LA	69.93	120.05	34.74	88.59	85.44	114.28	3.58	867	30	2021/8/13 0:00
刘杰	17	M	LA	38	115.2	7.59	74.15	66.99	139.16	11.2	842	39	2021/8/10 0:00
文志鹏	29	M	LA	107.9	156.59	66.29	151.73	75.17	200.94	0.98	834	28	2021/9/30
李秋	25	F	LA	41.37	143.02	26.89	108.82	63.99	160.83	10.4		26	2021/9/28
黄以满	27	M	LA	73.01	114.47	25.01	99.87	91.4	128.69	9.99		5	2021/9/6
黄昌珍	25	F	LA	45.18	152.74	51.91	143.24	82.62	171.62	14.1	52	2	2021/9/15
王冲	26	M	LA	76.6	110.61			94.96	116.66	13.9	760	90	2021/10/18 10:21
冯小康	28	M	LA	64.69	135.64			83.82	129.83	5.48	817	25	2021/10/16 17:00
孔德钦	39	M	LA	59.86	182.99			87.4	150.1	6.67	53.9	34	2021/10/11 11:39
谢远仙	26	F	LA	51.47	201.65	30.92	203.64	72.88	195.37	6.69			
肖晓文	27	F	LA	72.76	125.75			89.04	112.88	10.3	835	35	2021/10/22 17:02
冯威润	22	M	IA	53.37	240.36			0		43			2021/1/25 14:38
周依柔	22	F	IA	55.33	137.35			89.58	150.68	0		16	2021/1/28 11:04
方烨炜	28	M	IA	68.86	130.02			0		0	118	9	2021/3/3 11:04
胡思达	27	M	IA	65.11	152.58			89.87	104.15	0			2021/3/15 8:19
万丽红	40	F	IA	57.58	168.41			64.01	162.29	0	721	16	2021/3/16 10:22
罗发成	50	M	IA	64.1	154.81			87.9	140.29	0	64	2	2021/3/31 15:18
康少明	24	M	IA	58.02	143.6					0	122	8	2021/4/30 15:40
何细华	33	F	IA	58.41	161.31					2.56	598	26	2021/4/25 16:00
李明裕	29	M	IA	59.53	180.31					0			2021/4/23 22:01
顾娟娟	34	F	IA	75.1	125.97					0	89.7	2	2021/4/16 16:56
何向兰	29	F	IA	57.73	149.46			85.24	113.65	0	750	2	2021/5/26 16:17
胡南祥	41	M	IA	76.74	128.73			94.48	105.97	0	59.5		2021/5/14 8:53
舒玲	41	F	IA	57.13	125.46					0	206	4	2021/6/21 10:34
陈祖芬	33	F	IA	56.49	165.5	32.24	132.89			0			2021/6/11 10:52
史庆华	43	M	IA	59.44	153.32					0	45.4		2021/6/9 10:13
曾雪云	27	F	IA	60.13	110.95	52.05	121.98	82.1	103.72	0	positive		2021/6/3 15:53
陈怡锋	31	M	IA	45.39	172.66	33.14	137.26	71.37	150.37	0	806	16	2021/7/27 14:32
李柔霞	29	F	IA	57.64	102.36	62.29	103.67	73.29	94.16	0		2	2021/7/19 14:25
王思涵	15	F	IA	60.41	123.1	62.43	120.9	75.97	115.77	0		18	2021/7/15 12:07
薛智萍	33	F	IA	47.12	150.28	32.67	138.58	79.11	112.24	0		7	2021/7/12 20:02

蔡山山	28	M	IA	52.75	170.15	40.95	150.2	73.19	160.45	0		6	2021/8/24 0:00
施丽茜子	33	F	IA	60.53	129.74			87.25	131.5	0	41.8	3	2021/9/29
刘海月	43	M	IA	44.84	150.02	15.23	160.31			0			
常运粮	36	F	IA	40.64	124.84					0	41	2	2021/9/21
巫丽侠	40	M	IA	44.8	277.23	29.05	228.31			0			2021/9/18
陶小文	31	F	IA	46.17	147.81	36.2	139.59			0			2021/9/8
杨洁	48	M	IA	74.75	138.67			89.62	133.18	0	44.2	19	2021/10/18 8:18
李文娟	33	F	NC	55.78	116.1								2021/4/29 14:47
陈少辉	30	F	NC	66.03	129.16								2021/4/25 11:46
张德意	25	F	NC	58.86	192.38								2021/4/9 14:32
陶珊	27	F	NC	67.81	158.41								2021/4/8 14:51
公建伟	26	F	NC	43.22	155.32								2021/7/19 11:31
何书恒	24	F	NC	64.15	147.73								2021/9/25
许文芳	30	F	NC	46.35	137.83								2021/10/1 9:52
刘正龙	28	F	NC	47.12	205.24								7月10日
李明明	25	F	NC	54.17	133.31								7月10日
吴高云	27	F	NC	54.06	147.23								7月10日
石鹏	27	F	NC	64.86	119.8								7月10日
邬亮晓	29	F	NC	72.02	131.68								7月10日
李根	32	F	NC	55.84	139.44								7月11日
NN	32	F	NC	64.04	148.4								7月11日
程彬	34	F	NC	58.99	130.96								2021/7/18
林嗣弦	28	F	NC	54.9	180.8								2021/7/18
LL	26	M	NC	54.48	129.3								2021/7/18
贺守第	34	M	NC	67.64	125.87								2021/7/24
严佳倩	27	M	NC	68.94	136.92								2021/7/24
陈飞宇	27	M	NC	53.72	122.4								2021/7/24
QQ	28	M	NC	62.49	113.35								7月24日
徐明	26	M	NC	69.24	128.6								2021/8/7
李惠群	32	M	NC	43.17	163.24								2021/8/7
杨洁	31	M	NC	45.29	138.45								2021/8/7
卫杨雄	23	M	NC	48.39	229.79								2021/8/7
徐国生	30	M	NC	68.44	156.82								2021/8/7
宋房珠	28	M	NC	58.25	150.39								2021/8/7
刘孟茧	24	M	NC	70.31	125.33								2022/7/16

刘湘艳	27	M	NC	61.84	139.22			2022/7/16
王瑞敏	36	M	NC	56.11	118.12			2022年7月23日
魏新	31	M	NC	71.66	124.27			2022年7月23日
梁强	31	M	NC	60.14	144.61			2022年7月23日
姚望	37	M	NC	53.89	181.18			2022年7月23日
王姣	32	M	NC	49.13	113.51			2022年7月24日
冯书仑	41	F	NC	73.93	151.21			2022年7月24日
何兴鸿	42	F	NC	67.51	148.67			2022年7月24日
祝继锋	38	F	NC	63.99	147.69			2022年7月24日
曾闻君	42	F	NC	44.84	158.84			2022年7月24日
马萍	42	F	NC	75.38	111.61			2022年7月24日
肖平平	48	F	NC	47.48	191.78			2022年7月24日
李温青	39	F	NC	63.75	150.2			2022年7月24日
林耀山	42	F	NC	63.86	154.44			2022年7月30日
陈清秀	40	F	NC	53.58	165.51			2022年7月30日
唐伟	38	F	NC	55.96	151.16			2022年7月30日
张德胜	48	F	NC	55.91	176.63			2022年7月30日
马彪	41	M	NC	69.92	134.32			2022年7月30日
许欣欣	40	M	NC	51.85	130.93			2022年7月31日
雷婕	42	M	NC	46.03	113.93			2022年7月31日
何宝华	44	M	NC	43.18	152.96			2022年7月31日
吕宝艳	47	M	NC	60.7	141.6			2022年7月31日
徐伟	43	M	NC	71.37	151.42			2022年7月31日
林树俊	42	M	NC	64.11	155.44			2022/8/6
梁立庚	42	M	NC	77.36	144.39			2022/8/6
陈庚	45	M	NC	61.38	150.95			2022/8/6

AS, ankylosing spondylitis;
CRP, C-reactive protein; ESR, erythrocyte sedimentation rate ;FF, fat fraction;
SCB, Subchondral bone, BME, bone marrow edema, FM, fat metaplasia.
