# Supplementary material for: Chemical Shift-Encoded MRI of Bone Metabolic Markers in Ankylosing Spondylitis
Source: Dis Markers. 2022 Oct 13;2022:1846667. doi: 10.1155/2022/1846667 (PMC9584712; doi:10.1155/2022/1846667)
Supplement: Supplementary Materials — Table S1: overview of FF, R2∗ values and clinical data of 4 groups. Table S2–S5: FF and R2∗ value of each ROI in different 4 groups. [file 1846667.f1.zip › Table S2.pdf]

FF&R2value in Early Active Group from October 2020 to November 2021

name	parame	Different Regions Of SIJ_ROI												S2 Vertebral Body_ROI
		1	2	3	4	5	6	7	8	9	10	11	12	13
宁盼盼	FF（%）	55.41	64.12	15.25	53.8	73	26.25	71.54	87.1	65.2	60.7	50.6	49.71	68.48
	R2*	147.2	161.5	123.2	153	188	176.68	162.2	140.5	171.9	210.2	201.18	248.83	118
廖秋妹	FF（%）	47.33	43.39	41.41	59.3	46.6	71.41	41.43	58.11	70.76	56.18	52.24	54.34	45.93
	R2*	171.73	143.36	170	153	201	153.5	148.32	159.9	192.3	161.6	163.34	138.61	121.27
夏水兰	FF（%）	12.28	23.14	13.68	29.4	14.7	18.14	13.93	20	17.1	16.19	16.36	15.7	17.61
	R2*	85.31	105.89	91.18	79.8	105	81.41	133.34	90.28	107.2	91.69	115.61	98.1	84.29
杨小莉	FF（%）	60.74	62.36	73.76	74.3	47.7	60.31	78.26	71.19	55.53	55.24	56.4	64.11	29.78
	R2*	161.75	126.06	119.8	76.3	85.8	137.35	129.74	95.94	120.6	108	217.4	75.89	71.25
陈韵怡	FF（%）	72.12	68.84	70.67	72.4	87.5	69.44	75.19	68.72	63.61	66.12	69.44	66.03	45.84
	R2*	156.91	137.56	156.9	147	136	190.11	175.56	173	193.8	179	188.03	193.38	199.69
吴旭基	FF（%）	61.73	62.58	56.67	58.3	68.8	62.97	47.95	50.54	60.95	58.11	56	54.86	86.77
	R2*	120.72	152.28	114.4	150	128	135.49	188.52	157.9	140.1	179.1	178	134.23	125.67
陈庆鸿	FF（%）	72.52	96.72	73.09	85.9	88.9	94.23	81.76	91.56	89.08	83.89	75.4	84.04	67.08
	R2*	160.81	128.09	59.93	114	173	148.56	155.33	149.2	155.7	191.5	175.04	201.65	268.32
刘新火	FF（%）	56.28	69.35	21.23	76.7	43.5	49.96	36.96	87.26	30.52	70.57	29.96	75.73	64.87
	R2*	224.6	174.19	164.6	147	228	233.96	210.12	106.4	171.4	184.4	220.83	161.73	187.96
宋艺敏	FF（%）	46.91	53.5	51.91	38.3	53.3	54.31	57.6	56.88	39.57	37.73	45.22	45.81	44.62
	R2*	193.85	119.12	162.4	123	141	115.12	154.56	119.8	141.2	125.4	140.57	117.85	101.19
钟杰	FF（%）													
	R2*													
彭欲娴	FF（%）	62.85	53.56	43.96	52.9	63.1	48.76	46	62	52.07	41.74	49	61.64	45.44
	R2*	151.41	158.7	196.1	134	125	156.59	181.4	170	170.4	165.4	199.4	159.5	150.1
谭珊珊	FF（%）	49.75	85.18	43.96	83.4	48.7	64.66	48.48	66.3	44.44	50.07	59.93	54.26	37.29
	R2*	103.93	80.38	112.7	83.4	130	106.83	160.3	113	142.1	121.1	130.6	107.6	114.18
王慧敏	FF（%）	53.8	60.87	57.17	56.5	55.9	77.5	77.54	50.08	58.36	51	44.04	42.7	40.69
	R2*	130.68	129.74	164.7	111	180	166.81	172.42	518	184.2	151.7	208.32	162.89	185.46
祖靖	FF（%）	58.55	63.54	58.58	66.7	74.8	68.17	63.26	62.22	47.5	49.5	57.23	63.25	57.87
	R2*	129.2	133.15	130.3	122	140	104.44	167.04	132.2	173.9	137.5	191.69	106.6	132.7
卢健	FF（%）													
	R2*													

	宋梓闻	FF（%）	57.69	54.55	59.83	45.4	64.2	26.67	51.04	25.78	42.47	26.83	33.33	44.63	20.59
		R2*	132.97	163.24	175	189	134	139.27	216.15	151.3	186	187.3	186.07	288.67	177.7
	沈睦	FF（%）	57.9	83.03	63.07	67.5	73.8	72.13	73.3	92.5	66.93	56.19	70.91	70.28	48.97
		R2*	162.39	133.09	131.8	117	115	121.2	149.9	63.57	154.2	163.9	162.22	119.12	191.67
	刘正龙	FF（%）	66.18	79.32	74.33	64.4	66.2	79.32	74.33	86.15	73.8	73.2	73.27	62.64	49.06
		R2*	116.82	119.62	140.8	140	117	119.62	140.82	100.2	109.4	145.8	147.1	170.03	160.82
	阮美君	FF（%）	50.79	50.04	52.08	63.8	52	46.29	43	55.42	71.54	42.29	5.29	55.18	45.67
		R2*	178.67	175.54	149.7	129	136	137.62	171.23	163.1	141.2	139.1	90.42	144.91	111
	黄雨湘	FF（%）	42.88	43.47	150.7	54.3	32.8	46.43	48.57	57.47	28.33	28.57	56.87	53.06	39.13
		R2*	129.97	155.33	175.8	157	108	182.9	199.4	132.4	164.5	114.1	158.47	145.76	175.79
	王佳	FF（%）	57.79	62.55	55.68	67.7	52.6	54.52	45.73	56.72	59.52	60.87	52.64	57.54	59.88
		R2*	148.42	127.86	158.4	97.6	154	135.92	253.82	146.2	152.7	147.7	164.45	150.79	127.58
	冯桂萌	FF（%）	55.77	62.17	60.45	63.1	59.8	60.82	53.79	63.18	78.09	57.35	61.52	62.43	61.36
		R2*	146.27	137.91	139.6	127	150	130.59	154.04	118.3	133.6	137.8	155.13	126.35	111.27
早期急性:	郭冬琪	FF（%）	51.16	50.05	41.58	46.9	56.7	38.76	33.41	50.72	52.22	33.47	20.16	40.72	35.93
		R2*	125.32	128.79	134.2	135	142	157.84	163.06	104	128.1	158.2	108.68	121.39	136.7
早期急性:	刘维平	FF（%）	39.21	37.37	39.53	46.5	49.5	42.35	45.06	26.87	42.22	30.47	32.06	33.53	33.44
		R2*	177.94	127.95	153.5	142	154	161.41	172.72	93.42	143.1	150.4	188.28	135.95	153.33

FF&R2value in Early Active Group from October 2020 to November 2021

name	parameter	Bone Marrow Edema						Fat Mateplasia			
		Edema1	E2	E3	E4	E5	Average	Fat1	F2	F3	Average
宁盼盼	FF（%）	30.61	6.24	63.57			33.47333	87.04	86.48	92.76	88.76
	R2*	190.78	189.88	106.1			162.2533	148.74	149.52	151.88	150.0467
廖秋妹	FF（%）						#DIV/0!	79.4	87.79	79.32	82.17
	R2*						#DIV/0!	128.6	153.58	208.37	163.5167
夏水兰	FF（%）	11.73					11.73				#DIV/0!
	R2*	192.09					192.09				#DIV/0!
杨小莉	FF（%）							84.14	83.9	84.45	84.16333
	R2*	158.86	112.1				135.48	114.95	107.9	68.59	97.14667
陈韵怡	FF（%）	18.46	70.5				44.48	85.3	92.83	87.79	88.64
	R2*	170.58	160.92				165.75	162.39	151.11	133.36	148.9533
吴旭基	FF（%）	40.18	46.05	53.84			46.69	94.9	88.58	82.02	88.5
	R2*	101.3	115.03	121.38			112.57	97.38	90.48	99.75	95.87
陈庆鸿	FF（%）	47.96	52.33				50.145	96.33	92.48	93.26	94.02333
	R2*	145.91	184.72				165.315	135.31	117.81	117.92	123.68
刘新火	FF（%）	4.64	13.38	10.34	9.26		9.405	86.74	96.15		91.445
	R2*	166.32	182	169.52	198.74		179.145	114.74	82.35		98.545
宋艺敏	FF（%）	10.69	43.93				27.31	90.35			90.35
	R2*	111.81	126.41				119.11	142.83			142.83
钟杰	FF（%）										
	R2*										
彭欲娴	FF（%）	6.03	1.31				3.67				
	R2*	128.59	142.69				135.64				
谭珊珊	FF（%）	45.89					45.89				
	R2*	146.89					146.89				
王慧敏	FF（%）	27.19	21.67				24.43				
	R2*	149.57	151.76				150.665				
祖靖	FF（%）										
	R2*										
卢健	FF（%）										
	R2*										

宋梓闻	FF（%）	35.83	36.38	44.08			38.76333				
	R2*	170.5	182.62	163.3			172.14				
沈睦	FF（%）	40.9	51.47				46.185	92.5	83.4		87.95
	R2*	144.4	178.38				161.39	63.57	63.57		63.57
刘正龙	FF（%）										
	R2*										
阮美君	FF（%）	8.83	6.17				7.5	70.58	71.58		71.08
	R2*	94.17	99.42				96.795	157.12	185.62		171.37
黄雨湘	FF（%）	15.52	22.57	28	27.24	20.5	22.766	71.14			71.14
	R2*	158.57	229.9	112.73	119.52	106.36	145.416	77			77
王佳	FF（%）	40.5	14.84				27.67				
	R2*	143.5	194.5				169				
冯桂萌	FF（%）	13.96	18.22	36.55			22.91				
	R2*	83.84	157.83	131.73			124.4667				
郭冬琪	FF（%）	9.74	17.21				13.475				
	R2*	84	95.89				89.945				
刘维平	FF（%）	12.72	7.72	4			8.146667				
	R2*	124.83	103.5	185.61			137.98				
