# Supplementary material for: Chemical Shift-Encoded MRI of Bone Metabolic Markers in Ankylosing Spondylitis
Source: Dis Markers. 2022 Oct 13;2022:1846667. doi: 10.1155/2022/1846667 (PMC9584712; doi:10.1155/2022/1846667)
Supplement: Supplementary Materials — Table S1: overview of FF, R2∗ values and clinical data of 4 groups. Table S2–S5: FF and R2∗ value of each ROI in different 4 groups. [file 1846667.f1.zip › Table S3.pdf]

# FF&R2* value in Late Active Group from October 2020 to November 2021

| name | paramete | Different Regions Of SIJ_ROI | | | | | | | | | | | | S2 Vertebral Body_ROI |
|---|---|---|---|---|---|---|---|---|---|---|---|---|---|---|
| | | 1 | 2 | 3 | 4 | 5 | 6 | 7 | 8 | 9 | 10 | 11 | 12 | 13 |
| 吴国龙 | FF (%) | 53.56 | 58.17 | 56.59 | 64.28 | 65.39 | 66.62 | 61.75 | 69.79 | 57.92 | 54.44 | 40.96 | 51.84 | 50.52 |
| | R2* | 186.92 | 195.92 | 139.77 | 132.44 | 122.04 | 150.88 | 170.8 | 90.62 | 160.5 | 206.44 | 163.08 | 213.36 | 161.26 |
| 申三玉 | FF (%) | 70.81 | 70.24 | 30.61 | 51.59 | 66.97 | 83.16 | 26.22 | 31.12 | 71.93 | 72.91 | 31.48 | 43 | 54.19 |
| | R2* | 145.45 | 163.91 | 143.87 | 231.72 | 159.77 | 120.47 | 223 | 102 | 104.23 | 160.12 | 207.55 | 150.24 | 221.77 |
| 程思 | FF (%) | 81.08 | 89.24 | 80.53 | 91.66 | 86.5 | 95 | 85.29 | 71.65 | 87.58 | 94.83 | 72.65 | 58.5 | 43.17 |
| | R2* | 144.16 | 147.32 | 164.12 | 147.39 | 140.68 | 146.25 | 140.1 | 152.4 | 160.03 | 132.58 | 167.9 | 178.27 | 243.57 |
| 蔡钦童 | FF (%) | 62.39 | 59.61 | 57.33 | 64.79 | 57.17 | 68.33 | 43.3 | 67.32 | 66.53 | 56.27 | 77.87 | 57.59 | 66.74 |
| | R2* | 174.21 | 198.15 | 165.27 | 157.24 | 207.7 | 97.33 | 247.4 | 98.71 | 193.62 | 289.37 | 204.37 | 250.47 | 158.32 |
| 罗可美 | FF (%) | 54.44 | 72.38 | 42.71 | 55.22 | 71.33 | 72.79 | 41.88 | 52.96 | 45.83 | 40.43 | 48 | 41.81 | 44.7 |
| | R2* | 155.52 | 129.12 | 190.67 | 178.56 | 157.44 | 122.29 | 220.7 | 174.3 | 197.5 | 188.47 | 194.87 | 183.96 | 156.63 |
| 洪美凤 | FF (%) | 48.44 | 53.52 | 34.97 | 59.17 | 46.83 | 45.59 | 42.83 | 44.44 | 44.93 | 42.42 | 32.17 | 46.44 | 33.48 |
| | R2* | 167.41 | 135.67 | 136 | 130.88 | 137.96 | 132.96 | 196.9 | 179.6 | 171.63 | 183.54 | 221.75 | 185.19 | 195.78 |
| 肖亮 | FF (%) | 51.08 | 61.81 | 72.26 | 80.96 | 80.23 | 73.5 | 69.77 | 92.15 | 87.7 | 92.12 | 67.92 | 95 | 37.77 |
| | R2* | 175.96 | 148.35 | 118.43 | 101.48 | 92.15 | 127.53 | 221.3 | 89.69 | 126.26 | 72 | 290.8 | 93.65 | 177.54 |
| 叶和兴 | FF (%) | 71.24 | 91.66 | 79.59 | 80.88 | 89.06 | 70.38 | 60.04 | 75.98 | 86.81 | 68.15 | 86.09 | 74.48 | 66.29 |
| | R2* | 103.86 | 97.84 | 111.78 | 60.14 | 95.68 | 112.46 | 113 | 88.61 | 128.4 | 189.08 | 131.19 | 151.5 | 117.8 |
| 何联 | FF (%) | 57.2 | 56.09 | 53.74 | 62.2 | 74.06 | 63.06 | 67.54 | 81.34 | 61.6 | 58.94 | 82.06 | 65.34 | 44.03 |
| | R2* | 211.34 | 174.34 | 107.06 | 100.54 | 104.74 | 219.2 | 204 | 94.89 | 178.4 | 177.4 | 153.8 | 139.46 | 225 |
| 凌春强 | FF (%) | 58.93 | 77 | 63.58 | 57.17 | 75 | 71.26 | 58.12 | 75.37 | 76.52 | 75.74 | 67.57 | 74.57 | 60.08 |
| | R2* | 200.93 | 135.88 | 160.31 | 220.78 | 150.08 | 174 | 188.4 | 137.1 | 136.56 | 130.52 | 203 | 142.7 | 151.72 |
| 曾雪涓 | FF (%) | 159.33 | 62.97 | 61.5 | 61.04 | 56.393 | 68.43 | 55 | 56 | 58.59 | 64.88 | 54.83 | 68.77 | 54.13 |
| | R2* | 148.67 | 120.33 | 160.77 | 95.67 | 139.5 | 136 | 174 | 121 | 143 | 129.58 | 155.07 | 107.93 | 126.6 |
| 余琼花 | FF (%) | 48.96 | 72.3 | 59.85 | 66.19 | 50.59 | 61.44 | 53.43 | 61.35 | 53.5 | 54.85 | 48.83 | 38.85 | 60.56 |
| | R2* | 177.74 | 186.81 | 153.7 | 111.65 | 146.15 | 152.88 | 182.4 | 143.9 | 153.88 | 145.19 | 206.74 | 134.85 | 138.33 |
| 罗惠 | FF (%) | 76.47 | 87.33 | 63.83 | 73.74 | 74.67 | 74.42 | 88.7 | 92.7 | 92.04 | 54.07 | 72.52 | 54.22 | 61.7 |
| | R2* | 120.2 | 127.9 | 133.07 | 122.2 | 142.5 | 111.9 | 111 | 132 | 124.37 | 114.27 | 122.33 | 106.56 | 124.78 |
| 林鹏彬 | FF (%) | 62.47 | 74.72 | 87.32 | 77.58 | 70.09 | 67.83 | 72.7 | 60.3 | 68.47 | 55.89 | 87.72 | 54.09 | 54 |
| | R2* | 115.71 | 89.38 | 115.71 | 111.8 | 121.82 | 94.5 | 149 | 109 | 118.75 | 154.14 | 124.72 | 135.97 | 101.68 |
| 刘杰 | FF (%) | 26.97 | 40.6 | 51.13 | 51.12 | 22.84 | 5.2 | 47.2 | 56.9 | 33.03 | 6 | 69.48 | 45.44 | 39.33 |
| | R2* | 90.5 | 110.9 | 110.73 | 119.3 | 94.27 | 54.57 | 189 | 126 | 137.27 | 103.79 | 135.09 | 110.71 | 125.8 |

| | | | | | | | | | | | | | | |
|---|---|---|---|---|---|---|---|---|---|---|---|---|---|---|
| 文志鹏 | FF（%） | 55.43 | 54.41 | 587.56 | 54.83 | 65.74 | 67.76 | 59.69 | 65.74 | 75.67 | 65.12 | 69.73 | 73.15 | 52.5 |
| | R2* | 115.71 | 200.9 | 127.7 | 184.62 | 107.67 | 126.1 | 188.8 | 155.1 | 151.78 | 172.08 | 159.92 | 188.81 | 175.19 |
| 李秋 | FF（%） | 37.46 | 49.58 | 20.15 | 52.74 | 30.07 | 50.59 | 36.58 | 40.31 | 36.74 | 47.24 | 45.07 | 49.96 | 38.52 |
| | R2* | 126.19 | 150.85 | 120 | 191.85 | 97.45 | 133.31 | 189.1 | 99.69 | 140.41 | 128.38 | 193.15 | 145.88 | 89.76 |
| 黄以满 | FF（%） | 86.33 | 65.12 | 61.52 | 61.72 | 92.96 | 87.78 | 52.62 | 68.52 | 92.46 | 72.2 | 67.52 | 67.38 | 57.46 |
| | R2* | 90.37 | 154.38 | 146.48 | 127.84 | 85.09 | 124.93 | 145.2 | 88.76 | 94.19 | 94.52 | 121.91 | 100 | 144.92 |
| 黄昌珍 | FF（%） | 52.96 | 56.16 | 29.23 | 56.96 | 56.5 | 40.89 | 36.88 | 42.08 | 54.2 | 32.58 | 35.65 | 48.04 | 28.12 |
| | R2* | 152.23 | 157.92 | 129.65 | 150 | 158.96 | 136.85 | 200.9 | 128.5 | 154 | 136.08 | 145.77 | 182.04 | 121.88 |
| 王冲 | FF（%） | 70.73 | 66.35 | 82 | 70.35 | 75.85 | 73 | 85.93 | 73.03 | 69.63 | 80.7 | 78.88 | 92.71 | 51.26 |
| | R2* | 118.83 | 131.56 | 126.56 | 100.68 | 129.91 | 104.26 | 106.7 | 93.82 | 136.07 | 97.23 | 109.21 | 72.5 | 184.35 |
| 冯小康 | FF（%） | 57.7 | 56.24 | 66.31 | 57.23 | 60.83 | 58.7 | 67.7 | 62.73 | 77.83 | 78.55 | 71.3 | 61.2 | 37.48 |
| | R2* | 52.53 | 53.05 | 43.32 | 195.1 | 150.93 | 140.59 | 146.6 | 128.7 | 140.59 | 161.45 | 185.8 | 229 | 258.67 |
| 孔德钦 | FF（%） | 57.63 | 66 | 52.34 | 64.69 | 58.81 | 60.86 | 57.34 | 60.86 | 60.37 | 60.23 | 59.26 | 59.87 | 48.55 |
| | R2* | 192.8 | 167.57 | 182.07 | 208.24 | 208.48 | 189.79 | 179.8 | 175.1 | 155.5 | 163.77 | 187.52 | 185.3 | 184.5 |
| 谢远仙 | FF（%） | 58.67 | 59.006 | 58.27 | 67.68 | 37.3 | 56.88 | 46.8 | 71.67 | 19 | 55.8 | 23.97 | 62.57 | 27.7 |
| | R2* | 162.36 | 222.47 | 144.42 | 111.56 | 135.27 | 160.33 | 292.4 | 182.8 | 273.48 | 218.23 | 321.39 | 195 | 150.43 |
| 肖晓文 | FF（%） | 58.59 | 75.85 | 52.52 | 70.17 | 77.5 | 63.63 | 64.81 | 69.38 | 63.5 | 68.48 | 64.08 | 144.58 | 65.88 |
| | R2* | 103.92 | 157.37 | 161.63 | 85.37 | 123.71 | 145.17 | 134 | 97.08 | 155.59 | 135.88 | 67 | 142.29 | 125.92 |
| 林晓山 | FF（%） | 41.65 | 40.48 | 39.84 | 51.31 | 64.5 | 49.57 | 35.64 | 52.91 | 38.83 | 45.93 | 32.84 | 44.52 | 39 |
| | R2* | 145.48 | 121.57 | 169.36 | 144.08 | 129.62 | 163.43 | 169.8 | 148.7 | 141.67 | 152.85 | 162.12 | 151.47 | 137.48 |

# FF&R2* value in Late Active Group from October 2020 to November 2021

| name | paramete | Bone Marrow Edema | | | | | | Fat Mateplasia | | | | | |
|---|---|---|---|---|---|---|---|---|---|---|---|---|---|
| | | Edema1 | E2 | E3 | E4 | E5 | Average | Fat1 | F2 | F3 | F4 | F5 | Average |
| 吴国龙 | FF（%） | 18.52 | 7.78 | | | | 13.15 | | | | | | 0 |
| | R2* | 130.12 | 81.17 | | | | 105.645 | | | | | | |
| 申三玉 | FF（%） | 27.76 | 38.27 | 31.12 | 43.59 | | 35.185 | 93.66 | 94.66 | 95.47 | | | 94.59667 |
| | R2* | 101.78 | 119.5 | 102.03 | 130.79 | | 113.525 | 130.88 | 123.34 | 107.22 | | | 120.48 |
| 程思 | FF（%） | 47.16 | 38.9 | | | | 43.03 | 93.91 | 90.86 | 94.64 | | | 93.13667 |
| | R2* | 183.68 | 176 | | | | 179.84 | 126.15 | 135.92 | 118.95 | | | 127.0067 |
| 蔡钦童 | FF（%） | 7.67 | 3.22 | | | | 5.445 | 83.75 | 85.38 | | | | 84.565 |
| | R2* | 184.44 | 147.89 | | | | 166.165 | 109.25 | 111.69 | | | | 110.47 |
| 罗可美 | FF（%） | | | | | | | 96.23 | 87.83 | | | | 92.03 |
| | R2* | | | | | | | 116.3 | 112.13 | | | | 114.215 |
| 洪美凤 | FF（%） | 15.25 | 40.67 | 22.39 | | | 26.10333 | | | | | | |
| | R2* | 223 | 128.62 | 156.72 | | | 169.4467 | | | | | | |
| 肖亮 | FF（%） | | | | | | | 80.69 | 81.16 | 73.5 | | | 78.45 |
| | R2* | | | | | | | 96.76 | 81.48 | 127.23 | | | 101.8233 |
| 叶和兴 | FF（%） | 14.29 | 28.63 | 39.71 | | | 27.54333 | 94.06 | 91.03 | 92.66 | | | 92.58333 |
| | R2* | 105.63 | 101.27 | 155.67 | | | 120.8567 | 79.06 | 108.86 | 91.54 | | | 93.15333 |
| 何联 | FF（%） | 55.71 | 35.43 | 50.23 | | | 47.12333 | 82.59 | 86.94 | 88.28 | | | 85.93667 |
| | R2* | 121.37 | 105.37 | 107.23 | | | 111.3233 | 144 | 144.44 | 113.22 | | | 133.8867 |
| 凌春强 | FF（%） | | | | | | | | | | | | |
| | R2* | | | | | | | | | | | | |
| 曾雪涓 | FF（%） | 52.81 | 58.59 | | | | 55.7 | 77.11 | 82 | | | | 79.555 |
| | R2* | 129.12 | 143 | | | | 136.06 | 136.78 | 183.69 | | | | 160.235 |
| 余琼花 | FF（%） | 27.52 | 26.44 | 29.83 | | | 27.93 | | | | | | |
| | R2* | 175.61 | 136.92 | 98.13 | | | 136.8867 | | | | | | |
| 罗惠 | FF（%） | 53.17 | 38.86 | 46.015 | | | 46.015 | 89.17 | 94.5 | | | | 91.835 |
| | R2* | 113.5 | 118.57 | 116.035 | | | 116.035 | 82.83 | 112.17 | | | | 97.5 |
| 林鹏彬 | FF（%） | 33.78 | 35.7 | | | | 34.74 | 76.5 | 87.72 | 92.1 | | | 85.44 |
| | R2* | 86.91 | 90.26 | | | | 88.585 | 109.23 | 124.72 | 108.9 | | | 114.2833 |
| 刘杰 | FF（%） | 11.5 | 6 | 5.27 | | | 7.59 | 64.5 | 69.48 | | | | 66.99 |
| | R2* | 52.79 | 103.79 | 65.87 | | | 74.15 | 143.23 | 135.09 | | | | 139.16 |

| | | | | | | | | | | | | | |
|---|---|---|---|---|---|---|---|---|---|---|---|---|---|
| 文志鹏 | FF（%） | 63.38 | 69.19 | | | | 66.285 | 84.07 | 66.27 | | | | 75.17 |
| | R2* | 168.88 | 134.58 | | | | 151.73 | 169.52 | 232.35 | | | | 200.935 |
| 李秋 | FF（%） | 34.41 | 19.5 | 26.76 | | | 26.89 | 62.92 | 65.06 | | | | 63.99 |
| | R2* | 93.93 | 123 | 109.52 | | | 108.8167 | 159.15 | 162.5 | | | | 160.825 |
| 黄以满 | FF（%） | 18.81 | 28.22 | 28 | | | 25.01 | 87.96 | 93.04 | 93.19 | | | 91.39667 |
| | R2* | 111.48 | 95.48 | 92.65 | | | 99.87 | 106.7 | 121.56 | 157.81 | | | 128.69 |
| 黄昌珍 | FF（%） | 21 | 154.69 | 5.93 | 26 | 60.54 | 53.632 | 82.62 | 82.62 | | | 69.853 | 78.36433 |
| | R2* | 132.94 | 119.5 | 166.79 | 153.72 | 139.7433 | 142.5387 | 120.62 | 222.62 | | | 156.3805 | 166.5402 |
| 王冲 | FF（%） | | | | | | | 96.33 | 95.64 | 92.91 | | | 94.96 |
| | R2* | | | | | | | 103.77 | 100 | 146.21 | | | 116.66 |
| 冯小康 | FF（%） | | | | | | | 82 | 85.63 | | | | 83.815 |
| | R2* | | | | | | | 131.15 | 128.5 | | | | 129.825 |
| 孔德钦 | FF（%） | | | | | | | 87.4 | | | | | 87.4 |
| | R2* | | | | | | | 150.1 | | | | | 150.1 |
| 谢远仙 | FF（%） | 30.88 | 32.4 | 29.47 | | | 30.91667 | 69.5 | 77.3 | 71.85 | | | 72.88333 |
| | R2* | 200.09 | 139.93 | 270.9 | | | 203.64 | 181.63 | 180.87 | 223.61 | | | 195.37 |
| 肖晓文 | FF（%） | | | | | | | 88 | 90.07 | | | | 89.035 |
| | R2* | | | | | | | 104.38 | 121.37 | | | | 112.875 |
| 林晓山 | FF（%） | 18.19 | 12.26 | | | | 15.225 | | | | | | |
| | R2* | 160.88 | 159.74 | | | | 160.31 | | | | | | |
