# Supplementary material for: Chemical Shift-Encoded MRI of Bone Metabolic Markers in Ankylosing Spondylitis
Source: Dis Markers. 2022 Oct 13;2022:1846667. doi: 10.1155/2022/1846667 (PMC9584712; doi:10.1155/2022/1846667)
Supplement: Supplementary Materials — Table S1: overview of FF, R2∗ values and clinical data of 4 groups. Table S2–S5: FF and R2∗ value of each ROI in different 4 groups. [file 1846667.f1.zip › Table S4.pdf]

FF&R2* value in Inactive Group from October 2020 to November 2021

name	parameter	Different Regions Of SIJ_ROI												S2 Vertebral Body
		1	2	3	4	5	6	7	8	9	10	11	12	13
冯威润	FF（%）	52.79	47.84	42.48	51.56	67.38	53.67	58.5	57.12	50.24	47.88	59.92	50.48	53
	R2*	216.46	335.92	293.92	265.96	107	269	244.42	209.68	269.83	223.96	182.5	241.84	190.33
周依柔	FF（%）	55.67	49.08	52.21	52.29	55.83	47.17	65.16	52.38	75.57	46.96	73.28	38.71	3.6
	R2*	177.86	144.21	98.83	118.92	152.92	143.2	127.44	134.71	155.76	120.5	177.56	136.79	67.72
方烨炜	FF（%）	90.29	59.5	62.92	67	75.17	70.29	76.48	56.62	85.12	64.32	67.12	72.96	57.64
	R2*	101.04	140.5	141.6	159.48	90.33	114.5	154.96	132.52	109.2	156.96	139.04	120.19	141.32
胡思达	FF（%）	52.72	79.27	63.33	73.83	59.64	61.62	82.91	60.2	61.46	56.75	70.76	58.8	29.46
	R2*	170.08	118.59	156.354	118.3	158.22	174.9	156.17	135.04	139.62	173	169.6	161.12	182.79
万丽红	FF（%）	63.11	60.79	56.17	58.44	59.28	56.82	51.82	69.24	53.68	56.68	54.24	50.74	51.84
	R2*	186.06	172.42	124.33	162.94	169.72	143.1	187.35	167.88	181.58	150.42	174.05	201	170.74
罗发成	FF（%）	58.38	63.67	64.08	58.52	68.04	65.52	65.36	71.12	65.2	54.16	70.92	58.5	46.24
	R2*	156.62	142.5	115.16	179.84	106.36	138.1	149.76	121.04	158.96	214.96	155.08	221.08	210.2
康少明	FF（%）	54.06	54.69	53.83	59.05	59.89	56.42	62.84	57.97	61.16	55.05	57.8	63.42	47.82
	R2*	148.67	155.36	186.5	131.77	137.42	124.6	141.7	130	132.32	177.5	142.55	114.87	196.92
何细华	FF（%）	57.7	58.97	57.38	50.64	66.85	50.71	68.66	60.4	61.9	55.93	53.92	57.8	50.78
	R2*	154.96	176.73	180.62	74.43	181.17	144.7	180.71	153.27	184.39	173.71	183.57	147.46	169.73
李明裕	FF（%）	44	52.03	53.25	50.74	58.2	61.47	159.52	61.94	42.06	44.74	43.06	43.35	46.44
	R2*	210.75	195.18	162.47	173.5	154.1	131.6	154.06	139.52	239.44	196.26	216.06	190.77	186.71
顾娟娟	FF（%）	81.08	51.21	89.2	67.72	76.72	68.81	87.06	80.53	76.05	67.19	81.19	74.42	50.34
	R2*	127.37	113.71	107.5	127	123.69	111.2	115.64	107.74	158.03	158.53	148.81	112.39	140.03
何向兰	FF（%）	44	66.74	51.17	62.89	67.25	57.76	52.18	61.34	62.15	49.68	47.06	56.84	69.59
	R2*	122.71	142.38	169	148.06	142.28	157.4	150.68	123.97	134.76	123.29	181.12	171.16	142.41
胡南祥	FF（%）	62.43	97.87	60.6	93.88	66.19	90.53	73.71	86.96	61.9	76.12	67.81	68.56	61.96
	R2*	121.1	107.57	148.1	85.42	138.56	107	168.52	102.59	160.47	123.21	138.67	135.89	122.58
舒玲	FF（%）	45.77	54.89	53.8	53.2	55.37	48.17	66.8	79.63	60.49	47.97	51.89	67.57	61.49
	R2*	124.94	136.71	147.14	127.54	110.46	116.1	128.71	113	130.17	116.11	133.83	120.74	106.51
陈祖芬	FF（%）	50	46.71	46.94	55.89	45.17	45.51	45.57	52.31	49.37	44.49	45.69	150.26	56.29
	R2*	204.66	188.11	174.34	160.14	118.31	126.1	170.74	184.94	156.34	165.77	179.23	157.29	130.49
史庆华	FF（%）	53.26	54.89	55.17	70.51	61.94	68.83	52.06	74.57	51.69	53.74	62.03	54.54	48.8
	R2*	157.43	145.2	155.83	118.11	113.2	148	176.06	101.63	212.43	202.83	161.57	147.54	142.17
曾雪云	FF（%）	62.46	56.35	57.45	43.84	79.78	58.96	63.77	70.51	85.22	47.06	45.31	50.82	36.56
	R2*	130.31	94.04	117.98	92.37	122.18	105.9	116.4	109.76	97.51	95.57	154.04	95.35	114.18
陈怡锋	FF（%）	49.27	48.8	47.95	44.52	18.9	59.4	59.75	58.6	19.55	43.6	50.73	43.6	44.94
	R2*	157.69	180.85	184.8	222.09	141.23	141.3	175.35	143.1	155.86	164.04	202.96	202.67	185.94

李柔霞	FF（%）	54.87	80.9	51.09	54.23	57.02	49.04	40.32	55.84	65.11	61.94	66.67	54.62	82.05
	R2*	95.02	103.02	141.76	87.84	68.62	113.9	121.7	77.5	110.37	100.42	98.12	110.04	93.22
王思涵	FF（%）	62.93	64.74	77.89	60.16	70.59	19.74	70.52	56.58	66	45.77	71.74	58.22	52.08
	R2*	131.52	132.89	102.74	120.12	108.11	91.63	153.42	124.8	124.6	132.96	146.52	107.85	137.73
薛智萍	FF（%）	40.75	47.95	43.1	62.72	44.3	51.35	40.74	49.94	40.84	50.68	42.68	50.43	65.18
	R2*	141.86	139.95	169.1	100.1	161.6	139.2	166.93	160.91	151.57	141.39	178.49	152.27	113.3
蔡山山	FF（%）	42.32	54.36	45.87	54.22	51.76	57	53.81	60.03	47.95	57.54	56.44	51.7	35.13
	R2*	172.3	192.77	177.28	168.68	156.03	185	198.4	153	160.7	161.26	163.85	152.4	140.67
施丽茜子	FF（%）	55.55	49.04	54.65	60.54	84.26	73.7	54.39	66.35	70.07	50.15	52.69	55	46.92
	R2*	113.41	84.5	150.42	145.31	171.92	116.9	146.04	99.69	139.45	114.31	160.14	114.85	118.73
李丹	FF（%）	36.26	47.17	32.39	52.48	39.3	44.59	31.22	45.52	39.26	27.96	47.12	44.35	33.56
	R2*	118.37	109.7	143.87	112.36	122.48	136.7	125.35	92.19	131.91	135.64	149.46	120	117
常行君	FF（%）	59.24	40.6	41.04	63.5	48.67	40.56	31.69	53.7	41.88	42.3	27.52	46.93	43.6
	R2*	216.44	120.12	323.41	148.23	312.48	320.7	329.23	250.19	300.08	342.09	343.88	319.92	309.52
岳文琪	FF（%）	39.03	39.87	43.14	45.24	32.76	51.65	48.1	55.12	53	51.42	53.71	41.03	35.16
	R2*	180.27	147.03	155.55	120.3	143.3	184.1	174.97	135.58	166.84	58.87	145.23	161.59	140.74
王共海	FF（%）	72.06	78.03	74.63	81.93	67.69	79.27	71.88	73.41	89.27	67.91	68.03	72.83	57.8
	R2*	132.64	136.09	148.67	115.37	143.15	105.9	178.38	134.68	103.93	160.2	161.29	143.77	202.7

FF&R2* value in Inactive Group from October 2020 to November 2021

name	parameter	Bone Marrow Edema					Fat Mateplasia				
		Edema1	E2	E3	E4	Average	Fat1	F2	F3	F4	Average
冯威润	FF（%）										
	R2*										
周依柔	FF（%）						85.46	89.67	93.6		89.57667
	R2*						159.7	171.33	121		150.6767
方烨炜	FF（%）										
	R2*										
胡思达	FF（%）						84.61	94.21	90.79		89.87
	R2*						91.65	107.83	112.96		104.1467
万丽红	FF（%）						63.77	64.25			64.01
	R2*						147	177.58			162.29
罗发成	FF（%）						83.83	91.96			87.895
	R2*						162	118.58			140.29
康少明	FF（%）										
	R2*										
何细华	FF（%）										
	R2*										
李明裕	FF（%）										
	R2*										
顾娟娟	FF（%）										
	R2*										
何向兰	FF（%）						84.9	85.57			85.235
	R2*						117.7	109.6			113.65
胡南祥	FF（%）						96.58	97.87	89		94.48333
	R2*						112.42	107.57	97.93		105.9733
舒玲	FF（%）										
	R2*										
陈祖芬	FF（%）	32.24				32.24					
	R2*	132.89				132.89					
史庆华	FF（%）										
	R2*										
曾雪云	FF（%）	56.36	47.73			52.045	84.19	80			82.095
	R2*	119.73	124.23			121.98	91.44	116			103.72
陈怡锋	FF（%）	53.92	18.9	26.6		33.14	69.65	73.09			71.37
	R2*	141.27	141.23	129.29		137.2633	149.92	150.82			150.37

李柔霞	FF（%）							76.71	69.87			73.29
	R2*							85.31	103			94.155
王思涵	FF（%）	46.44	63	77.85		62.43		77.89	74.04			75.965
	R2*	117.26	119.26	126.19		120.9033		102.74	128.8			115.77
薛智萍	FF（%）	40.38	24.96			32.67		81.52	79.72	76.08		79.10667
	R2*	177.25	99.91			138.58		115.46	130.4	90.87		112.2433
蔡山山	FF（%）	46.9	35			40.95		75.76	70.62			73.19
	R2*	170.19	130.21			150.2		139.38	181.52			160.45
施丽茜子	FF（%）							92.38	82.12			87.25
	R2*							121.38	141.62			131.5
李丹	FF（%）											
	R2*											
常行君	FF（%）	39.7	18.4			29.05						
	R2*	262.83	193.78			228.305						
岳文琪	FF（%）	28.5	43.89			36.195						
	R2*	107.56	171.61			139.585						
王共海	FF（%）							88.24	91			89.62
	R2*							169.05	97.31			133.18
