# Supplementary material for: Chemical Shift-Encoded MRI of Bone Metabolic Markers in Ankylosing Spondylitis
Source: Dis Markers. 2022 Oct 13;2022:1846667. doi: 10.1155/2022/1846667 (PMC9584712; doi:10.1155/2022/1846667)
Supplement: Supplementary Materials — Table S1: overview of FF, R2∗ values and clinical data of 4 groups. Table S2–S5: FF and R2∗ value of each ROI in different 4 groups. [file 1846667.f1.zip › Table S5.pdf]

Normal control Group from October 2020 to August 2022

name	param	Different Regions Of SIJ_ROI											S2 Vertebral Body_ROI	
		1	2	3	4	5	6	7	8	9	10	11	12	13
李文娟	FF（%）	45.63	58.18	77.22	61	57.38	54.85	61.95	60.63	49.4	41.86	48.45	52.75	51.9
	R2*	133.74	91.73	127.29	86.25	133.57	104.56	127.17	89.15	141.05	129.11	132.57	96.97	99.22
陈少辉	FF（%）	51.64	82.36	70.64	88.95	69.85	67.05	57.58	71	60.2	61.9	55.67	55.48	53.48
	R2*	130.57	98.36	105.26	95.83	159.17	156.41	158.73	105.1	114.56	153.93	137.77	134.27	129.45
张德意	FF（%）	34.05	54.05	68.4	75.2	56.61	60.29	67.75	63.59	46.7	65.3	49.4	65.02	58.05
	R2*	228.1	179.59	163.57	174	205.63	181.43	171.14	175.1	246.89	167.57	252.74	162.93	217.67
陶珊	FF（%）	64.88	74.03	72.81	72.12	61.96	67.56	53.23	72.13	77.74	65.15	67.46	64.64	83.48
	R2*	134.36	108.78	41.77	150.3	214.19	158.85	189.46	143.2	113.07	202.77	211.77	232.44	115.96
公建伟	FF（%）	38.96	42.7	42.35	51.75	45.91	53.02	40.83	41.95	36.16	43.46	30.92	50.59	25.12
	R2*	133.07	163.78	159.88	142.9	150.46	134.74	173.44	153.8	174.16	156.89	162.77	157.98	155.18
Test03	FF（%）	54.59	68.63	62.97	67.53	56.66	65.45	65.1	72.41	52.03	74.44	54.13	75.83	51.79
	R2*	154.34	122.1	141.07	86.93	152.1	145.59	185.8	131.7	200.67	156.15	178.97	117.37	125.45
test04	FF（%）	40.23	50.28	44.54	52.35	49	40.95	46.16	54.52	43.39	41.6	44.04	49.08	46.48
	R2*	113.2	130.07	158.88	151.2	134.71	146.68	152	145.7	115.35	136.44	143.04	126.68	125.12
test05 （N	FF（%）	39.95	45.65	38.84	41.64	49.26	47.64	72.22	53.04	49.84	35.84	46.22	45.27	28.33
	R2*	214.91	243.78	210.88	220.3	202.83	190.2	152.09	167.2	189	224.44	213.17	234.12	122.88
test06	FF（%）	45.58	49.08	46.46	56.38	49.35	32.54	82.72	45.67	78.44	52.52	62.16	49.09	53.77
	R2*	145.96	138.84	163.96	99.42	155	146.92	114.76	132.8	123.4	126.04	148.8	103.78	133.77
test07	FF（%）	69.58	44.05	39.95	69.24	52.56	62.17	38.88	65.61	63.09	46.55	40.28	56.74	39.85
	R2*	120.5	155.32	165.18	138.4	131.24	169.78	162.54	122	176.39	142.68	142.64	140.09	103.62
test08	FF（%）	72.72	60.91	61.64	66.41	66.48	65	56.73	68	79.22	58.5	68.27	54.45	54.92
	R2*	80.32	136.68	117.32	114.1	108.52	108.77	149.23	103.5	71.39	146.28	122.64	178.91	140.68
test09	FF（%）	82.23	73.16	73.16	78.88	70.29	71.73	66.08	72.58	70.8	71.91	63.42	70	64.61
	R2*	111.96	149.82	116.68	120	130.62	148.14	120.75	92.54	145.2	152.68	163.71	128.13	162.75
test14	FF（%）	59.397	26	43.94	55.47	61.47	54.21	69.31	68.97	53.94	55.44	52.41	69.47	52.59
	R2*	139.53	144.06	167.94	123.5	156.91	125.67	133.14	124.4	138.11	116.35	154.59	149.03	148.25
test15	FF（%）	59.88	64.14	65.62	62.94	69.91	59.59	67.34	64.28	62.44	63.38	60.53	68.41	65.44
	R2*	158.94	136.94	152.24	142.1	155.31	173.06	144.09	138.8	150.44	147.32	161.75	119.84	187.76
test16	FF（%）	53.71	61.16	47.59	64.08	73.11	54.44	67.09	65.59	66.97	55.81	48.16	50.19	43.03
	R2*	146.85	117.25	148.5	83.25	103.83	125.92	130.32	125.1	142.75	131.06	175.56	141.09	133
test15	FF（%）	52	51.56	48.11	56.19	54.84	63.12	38.56	64.65	62.06	55.28	49.53	62.85	48.47
	R2*	228.86	188.74	214.14	170.2	154.16	161.24	223.15	111.6	161.06	168.61	224.5	163.38	217.25
test16	FF（%）	46.26	42.53	55.88	68.59	71	47.71	64.41	65.84	63.15	40.76	52.34	35.25	44.5
	R2*	164.56	93.18	148.16	113.2	103.19	131.79	129.24	108	135.64	131.38	155.12	138.25	134.29

test16	FF（%）	64.78	64.18	63.97	65.16	79.53	69.19	76.53	72.72	74.44	58.88	63.88	58.38	43.94
	R2*	108.39	160.47	150.97	157.2	92.26	101.78	112.58	107.3	95.19	156.59	132.28	135.47	153.7
test17	FF（%）	64.62	82.88	56.65	72.81	55.31	84.78	56.62	75.09	66.86	63.47	80.71	67.5	43.35
	R2*	135.47	110.78	157.85	124.5	146.375	115.66	172.06	114.7	131.56	155.62	155.65	122.85	147.71
test17	FF（%）	60	53.84	61.33	45.44	59.44	51.01	49.88	64.59	45.96	40.75	60.62	51.8	63.33
	R2*	72.19	116.56	113.04	191.1	131.84	177.93	142.88	97	134.56	158.88	47.46	85.44	181.83
Test18	FF（%）	53.68	54.352	55	53.24	63.25	74.78	80.92	67	69.68	62.62	58.48	56.92	46
	R2*	57.85	157.48	122.22	74.8	121.5	146.26	119.28	98.85	95.4	121.92	146.24	98.42	93.65
test18	FF（%）	68.75	70.22	77.59	63.29	78.38	74.22	69.24	69.81	74.06	63.28	60.06	62	44.72
	R2*	137.94	116.31	107.62	126	108.12	117.84	118.5	128.5	127.94	144.53	168.33	141.58	137.81
test19	FF（%）	35.92	39.88	35.88	65.25	43.85	45.41	40.84	47.94	39.47	37.64	38.72	47.21	30.25
	R2*	182.89	171.85	157.21	93.38	136.53	183.69	183.22	150.9	172	179.89	172.81	174.53	188.16
test20	FF（%）	40.33	55.62	36.62	55.56	45.71	45.36	46.14	52	39.75	44.38	37.09	44.88	52.85
	R2*	131.39	112.75	138.71	120	133.12	142.31	157.72	130.9	135.91	138.38	180.53	139.71	136.18
卫杨雄	FF（%）	42.02	52.15	44.22	67.18	50.41	45.62	45.00	68.43	38.67	45.64	38.86	42.45	49.67
卫杨雄	R2*	219.31	223.17	247.76	191.8	226.61	224.96	228.94	144.4	291.52	241.49	243.78	273.69	180.42
徐国生	FF（%）	61.49	74.23	73.85	77.81	64.76	72.27	62.9	66.98	61.98	75.55	56.14	73.26	66.17
徐国生	R2*	156.57	145.1	150.45	120.7	161.35	189.64	176.87	161.8	176	121.89	180.11	141.33	162.42
宋房珠	FF（%）	66.43	59.18	59.15	63.50	58.32	68.81	41.16	65	52.11	47.57	64.09	53.64	44.51
宋房珠	R2*	112.23	197.82	148.19	150.7	76.66	170.36	196.89	156.8	164.89	146.32	147.45	136.34	142.15
刘孟茧	FF（%）	64.5	72.64	56.76	77.2	76.00	80.16	67.34	77.96	66.43	68.77	65.16	70.8	64.14
刘孟茧	R2*	167.34	154.11	201.43	83.77	125.02	85.2	124.47	79.55	144.7	117.18	128.3	92.84	161.55
刘湘艳	FF（%）	59.85	66.91	63.43	73.1	52.77	62.19	55.21	67.68	60.68	59.89	59.43	60.89	59.51
刘湘艳	R2*	97.89	109.91	99.07	97.45	174.39	173.13	183.83	141.6	145.64	157.15	136.07	154.43	172.00
王瑞敏	FF（%）	55.8	71.43	54.57	54.67	57.02	68.02	61.14	72.30	31.89	50.55	34.05	61.93	51.64
王瑞敏	R2*	126.64	123.62	147.85	85.71	128.09	123.59	122.55	59.17	124.77	140.07	128.68	106.70	129.59
魏新	FF（%）	59.82	79.20	72.62	84.51	63.30	76.61	55.888	80.21	64.64	73.16	69.60	80.32	58.64
魏新	R2*	121.11	122.27	124.81	109.8	128.77	95.93	169.9	90.02	154.60	132.86	145.59	95.62	114.55
梁强	FF（%）	54.55	57.91	54.19	57.14	67.86	63.32	56.16	69.64	65.09	64.75	46.07	64.95	55.13
梁强	R2*	166.33	145.34	164.53	####	118.36	140.20	163.39	106.5	136.23	145.36	175.20	117.95	161.45
姚望	FF（%）	47.19	56.39	49.20	59.50	61.45	58.14	56.29	59.77	47.43	56.82	44.23	50.24	47.88
姚望	R2*	174.77	177.73	181.11	148.4	151.05	213.89	197.81	176.7	173.23	197.43	200.53	181.55	176.64
王姣	FF（%）	40.69	51.07	44.11	54.72	56.38	48.50	48.98	49.88	54.45	41.86	50.62	48.34	36.82
王姣	R2*	138.38	98.39	136.91	140.8	137.66	99.18	81.30	82.45	120.57	118.00	132.90	75.55	92.95
冯书仑	FF（%）	74.57	78.82	73.33	76.07	73.55	68.83	72.14	71.90	76.51	72.98	73.83	74.59	73.27
冯书仑	R2*	136.18	120.34	134.88	118.8	181.20	189.02	170.05	159.9	131.96	163.82	168.32	140.16	137.25
何兴鸿	FF（%）	79.00	65.29	70.70	66.59	59.34	66.12	56.95	74.38	69.74	67.14	74.45	60.36	66.52

何兴鸿	R2*	98.73	169.40	94.21	137.3	156.51	171.26	194.81	#####	129.32	141.39	165.98	176.89	139.02
祝继锋	FF（%）	59.07	64.45	53.36	75.02	59.72	78.06	63.29	71.93	55.18	65.5	50.91	71.34	41.90
祝继锋	R2*	132.89	192.15	154.66	124.2	138.45	100.47	162.10	#####	182.45	133.84	183.61	149.02	196.64
曾闻君	FF（%）	40.45	49.30	33.59	55.64	37.20	57.14	47.61	80.83	29.61	41.02	30.98	34.71	37.81
曾闻君	R2*	135.18	143.98	174.32	####	169.30	133.52	161.80	120.9	197.57	179.75	205.72	147.29	173.75
马丽萍	FF（%）	76.05	80.44	76.49	82.88	85.22	63.39	90.49	67.92	83.79	63.19	68.35	66.30	46.40
马丽萍	R2*	107.14	114.58	125.35	87.68	109.32	133.30	103.29	101.8	105.70	111.27	122.20	117.70	98.56
肖平平	FF（%）	41.88	54.38	38.20	67.12	46.91	38.36	45.47	57.05	39.98	47.43	43.72	49.25	37.87
肖平平	R2*	183.67	189.56	165.80	179.6	195.33	236.31	185.58	185.9	218.47	180.08	217.07	164.09	146.04
李温青	FF（%）	56.49	62.55	54.34	83.00	64.76	67.80	59.41	60.49	55.68	76.87	61.72	61.94	74.12
李温青	R2*	151.71	175.85	159.94	112.7	161.30	145.48	166.96	126.3	164.12	154.60	159.94	123.43	139.35
林耀山	FF（%）	77.58	65.77	65.47	71.13	68.17	70.08	57.35	54.56	71.53	49.12	56.65	58.92	38.47
林耀山	R2*	104.47	151.51	120.65	132.2	118.60	146.68	179.73	182.9	115.08	251.05	170.30	180.10	281.42
陈清秀	FF（%）	49.35	57.25	48.93	59.15	51	63.38	48.03	56.85	52.35	51.77	48.03	56.85	52.56
陈清秀	R2*	153.35	176.57	177.18	172	170.32	159.04	173.38	150.8	170.32	159.04	173.38	150.78	168.71
唐伟	FF（%）	51.24	58.78	53.68	65.72	54.53	65.20	50.92	61.74	43.70	53.67	56.96	55.40	52.35
唐伟	R2*	157.17	131.38	180.58	123.4	168.88	119.22	153.19	163.2	170.70	155.73	158.95	131.56	122.15
张德胜	FF（%）	54.68	44.68	49.62	61.90	57.10	63.43	52.65	59.06	60.73	67.78	46.28	52.97	53.02
张德胜	R2*	166.08	201.82	214.87	138.3	178.33	154.13	214.68	143.6	174.07	132.80	235.93	165.03	242.16
马彪	FF（%）	74.22	70.46	72.37	73.52	71.20	66.88	66.05	70.93	69.32	60.48	66.92	76.65	57.45
马彪	R2*	101.56	125.33	130.88	132	120.50	137.46	155.28	123.3	139.72	175.51	163.75	106.56	191.97
许欣欣	FF（%）	40.76	59.31	32.64	58.18	53.65	63.22	58.50	59.92	43.14	53.08	50.02	49.79	65.06
许欣欣	R2*	105	144.85	162.98	133	115.38	118.52	160.29	124.1	119.84	123.48	138.68	124.96	152.40
雷婕	FF（%）	40.43	62.87	46.27	55.29	44.25	65.10	38.78	51.67	35.53	36.32	38.02	37.84	42.31
雷婕	R2*	119.94	105.58	122.50	106.1	113.60	101.10	121.20	95.31	111.96	129.30	124.08	116.51	68.75
何宝华	FF（%）	41.99	50.86	23.87	49.83	48.32	45.08	47.18	48.76	46.83	39.85	36.50	39.13	37.40
何宝华	R2*	156.1	138.41	150.33	154.6	154.64	146.46	161.36	156.5	145.54	147.88	157.73	165.98	143.72
吕宝艳	FF（%）	69.16	62.78	52.35	64.86	56.52	60.96	55.56	59.82	72.92	56.23	60.06	57.21	43.58
吕宝艳	R2*	130.49	129.51	141.04	101.2	151.76	148.07	148.58	172	149.83	138.34	148.21	140.21	95.07
徐伟	FF（%）	73.85	74.82	79.59	79.06	66.87	76.48	59.03	76.03	77.29	69.72	50.42	73.32	63.41
徐伟	R2*	108	171.55	118.31	135	152.72	145.00	185.39	#####	132.65	189.90	213.68	146.68	162.24
林树俊	FF（%）	67.1	71.97	68.38	62.00	70.29	59.67	78.55	69.70	63.14	57.32	54.55	46.68	52.52
林树俊	R2*	114.1	111.21	116.79	115.6	111.67	210.24	170.61	124.6	142.66	223.39	182.79	241.61	182.42
梁立庚	FF（%）	83.48	70.72	78.93	74.06	74.00	73.36	74.97	80.82	74.29	84.42	78.00	81.31	82.58
梁立庚	R2*	117.42	139.86	157.24	124.4	187.79	147.45	156.85	161.6	164.71	115.06	136.83	123.45	89.36
陈庚	FF（%）	55.77	62.87	59.12	59.33	65.81	67.79	62.55	70.13	56.17	58.74	57.65	60.67	42.24
陈庚	R2*	165.84	133.77	114.94	155.8	154.19	145.45	143.86	116.1	167.17	147.94	186.68	179.58	180.86
